# Deposition Process and Properties of Electroless Ni-P-Al_2_O_3_ Composite Coatings on Magnesium Alloy

**DOI:** 10.1186/s11671-018-2608-0

**Published:** 2018-07-06

**Authors:** Rong Hu, Yongyao Su, Yurong Liu, Hongdong Liu, Yingmin Chen, Changsheng Cao, Haitao Ni

**Affiliations:** 1 Research Institute for New Materials Technology, Chongqing University of Arts and Sciences, Chongqing, 402160 People’s Republic of China; 2 College of Materials and Chemical Engineering, Chongqing University of Arts and Sciences, Chongqing, 402160 People’s Republic of China; 3 Daye Nonferrous Metals Group Holdings Co., Ltd., Huangshi, 435005 People’s Republic of China

**Keywords:** Magnesium alloy, Nano-Al_2_O_3_ particles, Ni-P-Al_2_O_3_ composite coatings, Deposition process, Property

## Abstract

To improve the corrosion resistance and wear resistance of electroless nickel-phosphorus (Ni-P) coating on magnesium (Mg) alloy. Ni-P-Al_2_O_3_ coatings were produced on Mg alloy from a composite plating bath. The optimum Al_2_O_3_ concentration was determined by the properties of plating bath and coatings. Morphology growth evolution of Ni-P-Al_2_O_3_ composite coatings at different times was observed by using a scanning electronic microscope (SEM). The results show that nano-Al_2_O_3_ particles may slow down the replacement reaction of Mg and Ni^2+^ in the early stage of the deposition process, but it has almost no effect on the rate of Ni-P auto-catalytic reduction process. The anti-corrosion and micro-hardness tests of coatings reveal that the Ni-P-Al_2_O_3_ composite coatings exhibit better performance compared with Ni-P coating owing to more appropriate crystal plane spacing and grain size of Ni-P-Al_2_O_3_ coatings. Thermal shock test indicates that the Al_2_O_3_ particles have no effect on the adhesion of coatings. In addition, the service life of composite plating bath is 4.2 metal turnover, suggesting it has potential application in the field of magnesium alloy.

## Background

Magnesium (Mg) alloys have attracted a great deal of attention and scientific research, owing to low density, high specific strength, and excellent machinability [1, 2]. Therefore, Mg alloys are usually utilized in aerospace, electronics, and automobile fields [3, 4]. However, the application of Mg alloys has been limited on account of the undesirable defects in anti-corrosion and wear resistance [5, 6]. Thus, surface anti-corrosion and anti-friction methods, such as micro-arc oxidation film, chemical conversion coating, thermal spraying, physical vapor deposition, electroplating, and electroless plating, have been developed for Mg alloys [7–13].

Electroless nickel-phosphorus (Ni-P) plating is one of the most effective surface technology for Mg alloys, since it has excellent comprehensive advantages in low-cost, efficient, corrosion resistance, and wear resistance [14, 15]. Therefore, electroless Ni-P coating plays an important role in the anti-corrosion field of Mg alloys. To further improve the performance of the Ni-P coating, nanoparticles, for instance, SiC, ZrO_2_, TiO_2_, SiO_2_, and Al_2_O_3_, etc. are usually added into electroless plating bath to prepare Ni-P nanoparticle composite coatings [16–20]. According to previous studies [20–23], the performance of the Ni-P coating is effectively improved by nanoparticles. Although the Ni-P nanoparticle composite coatings have relatively high performance compared with the Ni-P coating, there are three problems that have to be noted. Firstly, nanoparticles are easy to aggregate and form the active center in the electroless plating bath, which reduces the stability of plating solution. Secondly, process parameters of composite plating bath usually determine the content and distribution of nanoparticles in the coatings, and they are also key factors for improving the properties of coatings. Thirdly, the process of nanoparticle co-deposition with Ni-P is another influence factor on coating properties. Hence, these factors are worth the attention. Nano-Al_2_O_3_ particles are a cheap abrasive, which have high hardness and good chemical stability [24, 25]. It can be dispersed in the electroless nickel plating bath well. Therefore, Ni-P-Al_2_O_3_ composite coatings are usually employed as anti-corrosion and anti-wear coatings to protect steel or copper substrate. However, only a few reports focused on the electroless Ni-P-Al_2_O_3_ plating on magnesium alloy substrate [20, 22, 26]. Moreover, the study of the growth process of the Ni-P-Al_2_O_3_ coating on Mg alloys and the stability of composite plating bath is rather rare. Therefore, more details about the performance of composite bath and co-deposition process of Ni-P-Al_2_O_3_ need to be studied.

In the present work, to further enhance the properties of the Ni-P coating on Mg alloy substrate, we employed nickel sulfate and lactic acid system as the main salt and complexing agent, respectively, in the plating bath. Meanwhile, nano-Al_2_O_3_ powder was added into the electroless Ni-P plating bath. To obtain a suitable electroless composite plating bath for AZ91D Mg alloy, the process parameters of this bath were evaluated by deposition rate and coating properties. Furthermore, periodic cycle test was carried out to evaluate service life and stability of the plating bath at the optimum process conditions. To study the effect of nano-Al_2_O_3_ particles on the growth process of the coatings, the deposition behavior and phase structure of the Ni-P coating were discussed. In addition, the properties, including corrosion resistance, micro-hardness, and adhesion of coatings, were analyzed base on morphology and structure. The results showed that the properties of the Ni-P-Al_2_O_3_ composite coatings were preferable to that of the Ni-P coating, and electroless composite plating bath had good stability in service life. Therefore, our results in this work are a useful reference for the application of electroless Ni-P nanoparticle composite coatings on Mg alloy.

## Methods

### Preparation of the Composite Coatings

In this work, AZ91D die-cast Mg alloy with a size of 2 cm × 1 cm × 0.5 cm was employed as experimental material, which contains chemical composition in wt%: 8.5 Al, 0.34 Zn, 0.1 Si, 0.03 Cu, 0.002 Ni, 0.005 Fe, and 0.02 other and balance Mg. The AZ91D substrate was successively polished with no. 500 and 1000 SiC paper, rinsed with deionized water, and immersed in alkaline solution for 5 min at 65 °C, followed by acid pickling in a chromic acid solution (CrO_3_ 200 g/L) for 60 s. After that, the Mg alloy substrate was immersed in a hydrofluoric acid solution with a concentration of 380 mL/L for activation treatment about 10 min. The Mg substrate was cleaned with deionized water at each step. The basic bath composition and operation conditions of electroless nickel plating for magnesium alloy were illustrated as follows: 35 g/L NiSO_4_⋅6H_2_O, 35 g/L lactic acid, 30 g/L Na_2_H_2_PO_2_⋅H_2_O, 10 g/L NH_4_HF_2_, 3 mg/L stabilizing agent, pH 4.5~7.0, and temperature 70~90 °C. The electroless plating bath was kept in a glass beaker, which was placed in a thermostat-controlled water bath. A digital display electric stirrer was used to provide stirring force. The average particle size of the nano-Al_2_O_3_ particles is about 50 nm. The nano-Al_2_O_3_ particles were adequately dispersed in the bath under the ultrasonic wave condition before electroless plating.

### Tests for Deposition Rate and Stability of Plating Baths

To study the effect of nano-Al_2_O_3_ particles on the deposition rate of electroless nickel plating bath, the deposition rate is expressed in Eq. (1).

1 $$ v=\frac{\Delta w\times {10}^4}{\rho St} $$

where *v*, *ρ*, *S*, *t*, and △*w* represent deposition rate (μm/h), density of the Ni-P coating (~ 7.9 g/cm^3^), surface area of the Mg substrate (cm^2^), deposition time (h), and coating weight (g), respectively. In addition, the content of nano-Al_2_O_3_ particles in the coating was estimated by weighing method using an electronic balance (AR2140, Ohaus). To evaluate the stability of electroless plating bath, periodic cycle test (or metal turn over, MTO) was employed to evaluate the service life and stability of bath. Here, 1 MTO has defined that deposition weight of Ni is equivalent to the initial concentration of Ni^2+^ in the bath. Taking 1 L of plating bath as an example, about 7.8 g Ni is obtained from the bath $\left({C}_{{\mathrm{Ni}}^{2+}}=7.8\kern0.5em \mathrm{g}/\mathrm{L}\right)$ regarding as 1 MTO. In addition, a fresh mixture solution (${\mathrm{Ni}}^{2+}:{\mathrm{H}}_2{\mathrm{PO}}_2^{2-}=1:3$ in mole ratio) was added into the plating bath when the bath had a low deposition rate. The stability test was ended until the decomposition of plating bath. Thus, the expression of MTO can be presented as Eq. (2).

2 $$ \mathrm{MTO}=M/m $$

*M* and *m* represent the cumulative deposition weight of Ni and the concentration of Ni^2+^ in the plating bath, respectively.

### Materials Characterization

The surface morphology of the coating was observed by using a scanning electron microscopy (SEM, Hitachi S-4800). The structure of the coating was studied by the X-ray diffractometer (XRD, D/Max-2200, Japan) with a CuK_*α*_ radiation (*γ* = 0.154 nm).

### Electrochemical Measurement

A potentiodynamic polarization test was performed on an electrochemical analyzer (CHI800, Chenhua, China). Electrochemical experiment was carried out in a 3.5 wt% NaCl aqueous solution by using a classic three-electrode configuration, which consisted of a working electrode (sample, 1 cm^2^), a counter electrode (platinum), and a reference one (saturated calomel electrode). During the potentiodynamic sweep experiment, the sample was first immersed in the electrolyte solution for 30 min to stabilize the open circuit potential (*E*_0_). Tafel plot was transformed from the recorded data, and the corrosion current density (*i*_corr_) was determined by extrapolating the straight-line section of the anodic and cathodic Tafel lines. The experiment sweeping rate was 5 mV/s and was performed at 25 °C. The micro-hardnesses of the magnesium alloy with various composite coatings were evaluated by using a HXD-1000 micro-hardness tester with a Vicker indenter at a load of 100 g and durable time of 15 s. Thermal shock test was carried out to evaluate the adhesion of coatings [23]. It was described as follows: in an air atmosphere, the Mg substrate with Ni-P coating or Ni-P-Al_2_O_3_ coating was placed in a high-temperature box resistance furnace and heated to 250 ± 10 °C by a heating rate of 20 °C min^−1^ then quenched in a cold water. This process was repeated 20 times.

## Results and Discussion

**Fig. 1:**
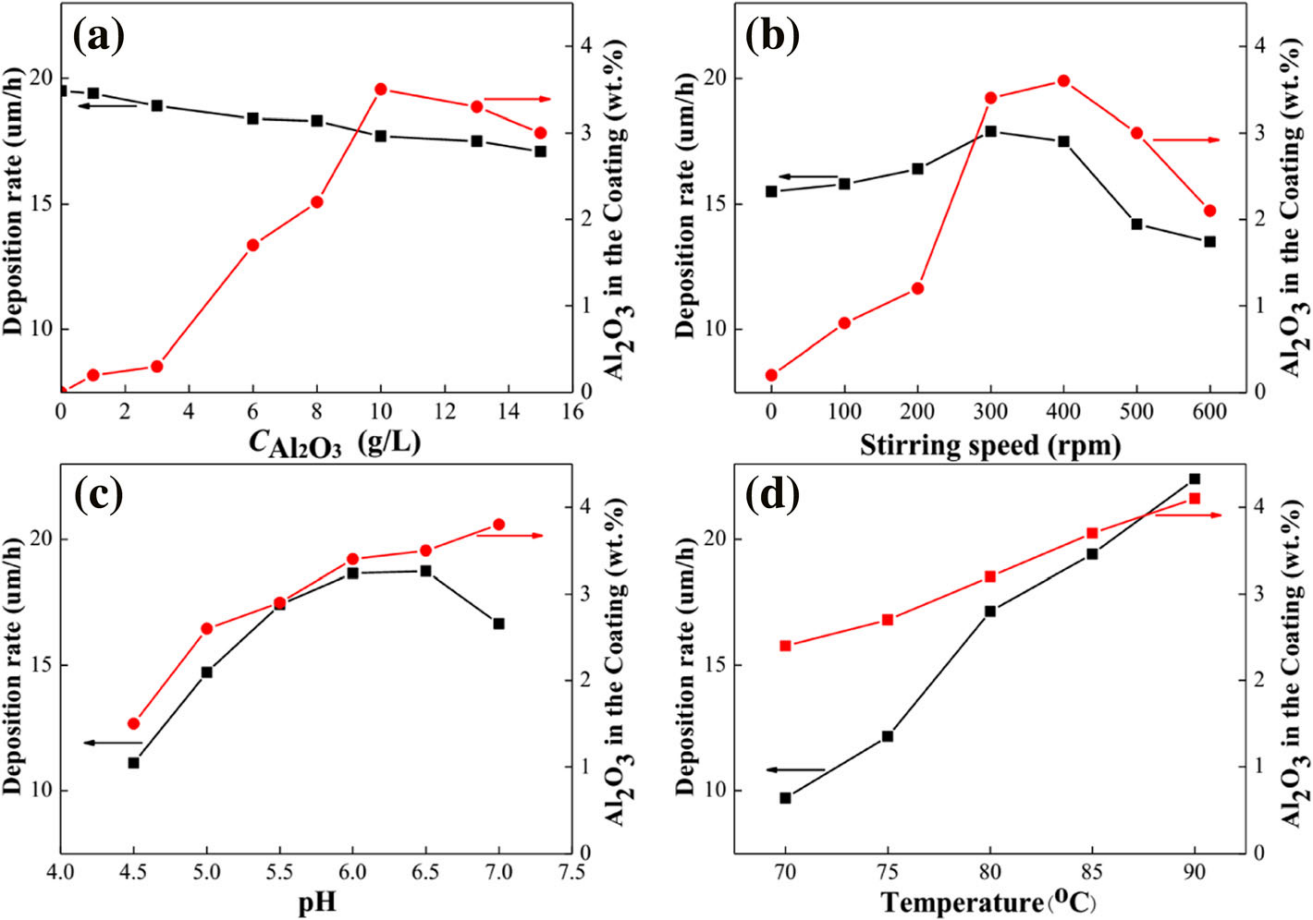
Effects of process parameters on deposition rate and Al_2_O_3_ content of coatings

Figure 1 shows the effects of the concentration of nano-Al_2_O_3_ particles, stirring speed, pH and temperature on the deposition rate, and Al_2_O_3_ content of composite coatings. As seen in Fig. 1a, the deposition rate has a slight decrease with the increase of Al_2_O_3_ particle concentration from 0 to 15 g/L. On the other hand, the Al_2_O_3_ content of the composite coatings gradually increases while the concentration of Al_2_O_3_ particles increases from 0 to 10 g/L. However, it decreases instead when the concentration of Al_2_O_3_ particle is higher than 10 g/L. This change is due to the aggregation of particles at high concentrations, which weakens the co-deposition behavior of Ni-P with Al_2_O_3_. In Fig. 1b, when the stirring speed is set at 300~400 rpm, the deposition rate and the Al_2_O_3_ content of composite coatings are 18 μm/h and 3.6%, respectively. The results demonstrate that dispersivity of Al_2_O_3_ particle in the plating bath is best at this range of stirring speed. As for acidic composite plating bath, the effect of pH value on the deposition rate and Al_2_O_3_ content of coatings is shown in Fig. 1c. The maximum deposition rate is up to 18.5 μm/h when the pH value is within the range of 6.0~6.5, whereas the Al_2_O_3_ content of coatings almost increases with pH value. Figure 1d shows that both the deposition rate and the Al_2_O_3_ content of composite coatings increase with temperature, since the activity of ions and particles is improved, and the reaction rate of the composite bath is also accelerated at high temperature. However, the stability of plating bath and the porosity of coatings become worse at high temperature (> 85 °C) [13, 22]. Based on the above analysis results, the preliminary process parameters and operating conditions of the composite bath are determined for magnesium alloy, that is, 35 g/L NiSO_4_⋅6H_2_O, 35 g/L lactic acid, 30 g/L Na_2_H_2_PO_2_⋅H_2_O, 10 g/L NH_4_HF_2_, 10 g/L nano-Al_2_O_3_ particles, 3 mg/L stabilizing agent, pH = 6.0~6.5, *T* = 85 °C, and stirring speed at 350 rpm.

**Fig. 2:**
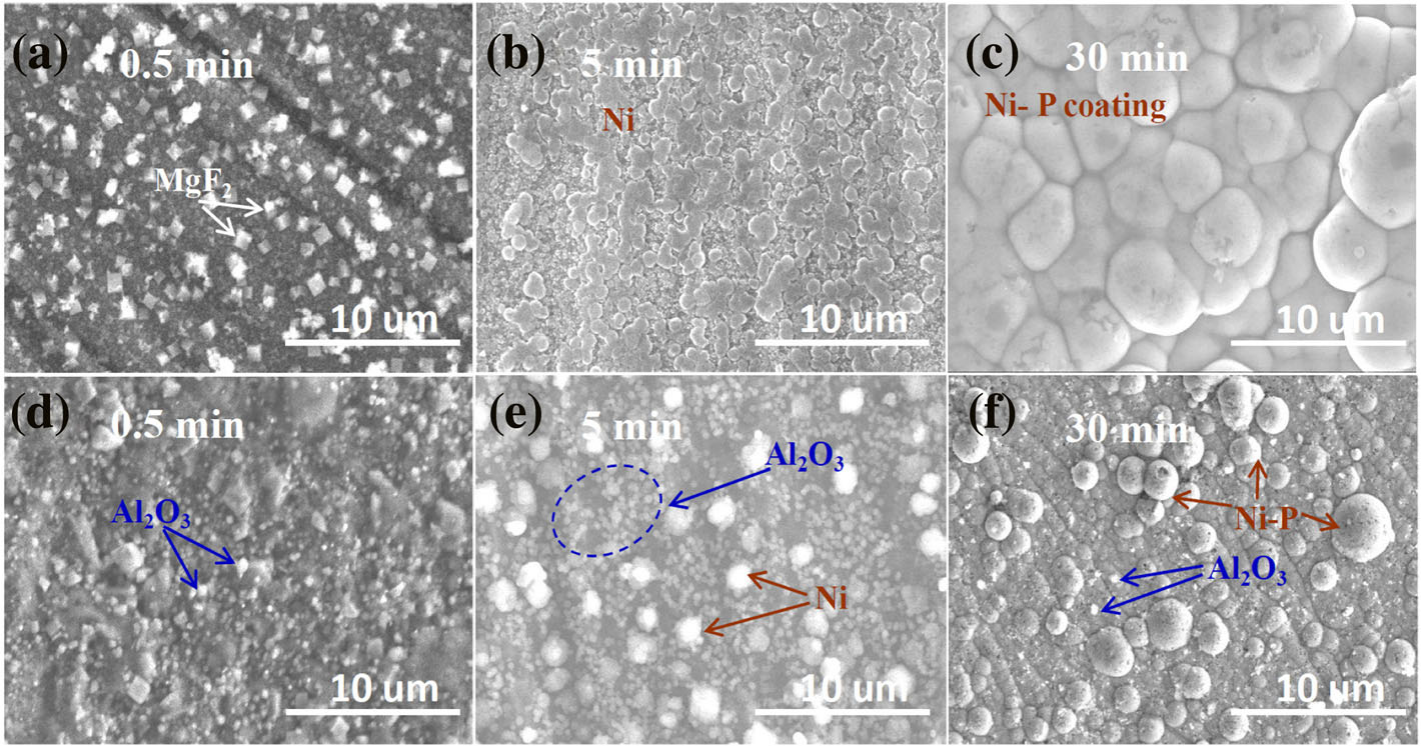
Surface morphology of Ni-P coating (top, **a**-**c**) and Ni-P-Al_2_O_3_ composite coatings (bottom, **d**-**f**) at different deposition times

To investigate the deposition process of Ni-P-Al_2_O_3_ coatings, the change of surface morphology images of magnesium alloy with deposition reaction time is shown in Fig. 2. For comparative analysis, Fig. 2a–c represents the deposition process of Ni-P coating, while Fig. 2d–f shows the co-deposition process of Ni-P-Al_2_O_3_ (3.6 wt%) composite coatings. Figure 2a is the morphology image of Mg substrate immersion in the plating bath for 0.5 min, a large number of cubic structure particles distributed on its surface. These cubic particles are confirmed as MgF_2_, which mainly forms in HF activation process, in consistent with the literature reports [23, 27]. However, the morphology of the image of Fig. 2d is distinctly different from Fig. 2a. The main difference shows that the MgF_2_ particles in Fig. 2d are less than that in Fig. 2a. In addition, many nano-Al_2_O_3_ particles are observed on the surface of Mg substrate. The change of morphology originates from Al_2_O_3_ particles that continuously impact on the surface of magnesium alloy at high temperature and stirring process. When the electroless Ni-P plating time is up to 5 min, as seen in Fig. 2b, Ni particles gradually grow and then cover the whole surface of magnesium alloy. But for electroless composite plating (see Fig. 2e), the larger Ni particles and nano-Al_2_O_3_ particles are observed on the surface of magnesium alloy, and the Ni-P-Al_2_O_3_ coatings do not completely cover the Mg substrate within 5 min. It indicates that the growth rate of Ni-P-Al_2_O_3_ coatings in the composite bath is lower than that of Ni-P coating in the bath without Al_2_O_3_ particles. This is an evidence to support the cause of the low deposition rate in the composite plating bath. When the electroless plating time is carried out for 30 min, the morphology of Ni-P coating and Ni-P-Al_2_O_3_ coatings is shown in Fig. 2c, f, respectively. As for Ni-P coating, the surface presents a dense and nodular structure with an average size of 3 μm. But in Fig. 2f, the averaged nodular size of Ni-P-Al_2_O_3_ composite coatings is apparently smaller than that of Ni-P coating. Moreover, it can be clearly observed that the nano-Al_2_O_3_ particles embed in Ni-P coating. Importantly, from the view of the surface distribution of Al_2_O_3_ particles, the distribution of Al_2_O_3_ particles in Fig. 2f is significantly less than that in Fig. 2c, e. This result indicates that deposition of Ni-P is dominant, while the deposition of Al_2_O_3_ particles becomes subordinate after a deposition reaction time of 5 min. Similar inferences also can be acquired from the relative content of Al_2_O_3_ particles in the coatings (Fig. 1). In other words, the effect of Al_2_O_3_ particles on the deposition process is mainly present in the initial stage of electroless nickel plating.

**Fig. 3:**
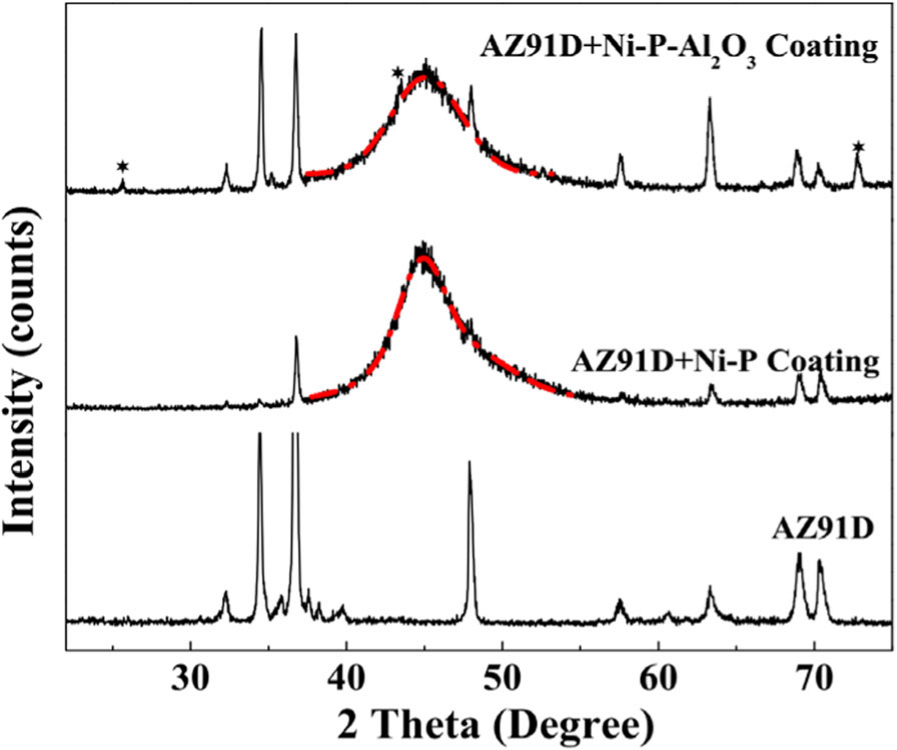
XRD patterns of the AZ91D substrate, Ni-P coating, and Ni-P-Al_2_O_3_ (3.6 wt%) composite coatings

To explore the effect of nano-Al_2_O_3_ particles on the structure of Ni-P coating, the XRD patterns of the AZ91D Mg alloy, Ni-P coating, and Ni-P-Al_2_O_3_ composite coatings are analyzed in Fig. 3. As seen in Fig. 3, the diffraction angle of crystal planes of magnesium alloy mainly concentrates in the range of 30°~70°, for instance, *α*(10$\overline{1}$0) 32.2°, *α*(0002) 34.2°, *β*(10$\overline{1}$1) 36.8°, etc. As for Mg alloy coated with Ni-P coating, the diffraction pattern of Ni-P coating exhibits a broadening peak and high-intensity diffraction at 44.7° that can be ascribed to the (111) crystal plane of a face-centered cubic (fcc) phase of nickel (Table 1) [28]. Moreover, the existence of such broad peak indicates the formation of Ni-P coating with a mixed amorphous crystalline structure. After plating the Ni-P-Al_2_O_3_ (3.6 wt%) composite coatings, three new diffraction peaks can be evidently found at 25.6°, 43.5°, and 73.2°. These peaks are attributed to the characteristic diffraction peaks of Al_2_O_3_ compared with the PDF card no. 88-0826. Hence, Ni-P-Al_2_O_3_ composite coatings are deposited on the surface of Mg alloy. In addition, the diffraction peak of the (111) crystal plane of Ni shifts to 45.2° (see Table 1) in Ni-P-Al_2_O_3_ composite coatings, suggesting nano-Al_2_O_3_ particles have a certain influence on the (111) crystal plane spacing of Ni. According to Bragg formula, *nλ* = 2*d*sin*θ* (*n* = 1, 2, 3, ..., *λ =* 0.154 nm, *d* and *θ* represent interplanar spacing and diffraction angle, respectively), the (111) crystal plane spacing of Ni is reduced about 3% by Al_2_O_3_ particles. Furthermore, both the (111) diffraction peaks of Ni in the Ni-P coating and Ni-P-Al_2_O_3_ composite coatings were fitted by Gauss function, respectively. The result shows that the full width at half maximum (FWHM) of this diffraction peak in Ni-P-Al_2_O_3_ composite coatings is broader than that in Ni-P coating (Table 1). According to Scherrer formula, *D* = *Kγ*/*B*cos*θ* (*D*, *K*, *B* represent crystalline grain, Scherrer constant, and FWHM, respectively), the crystalline grain of Ni-P-Al_2_O_3_ composite coatings is reduced about 8% by Al_2_O_3_ particles compared with Ni-P coating. This implies that nano-Al_2_O_3_ particles refine the size of Ni crystalline grain, which is consistent with the observed result of SEM above.

**Table 1:** The characteristic parameters of diffraction peak of Ni-P (111) in the Ni-P coating and Ni-P-Al_2_O_3_ (3.6 wt%) coatings

Coatings	Intensity	Peak	FWHM
Ni-P coating	1424.7	44.7 ± 0.01	4.97
Ni-P-Al_2_O_3_ coating	1205.4	45.2 ± 0.01	5.36

**Fig. 4:**
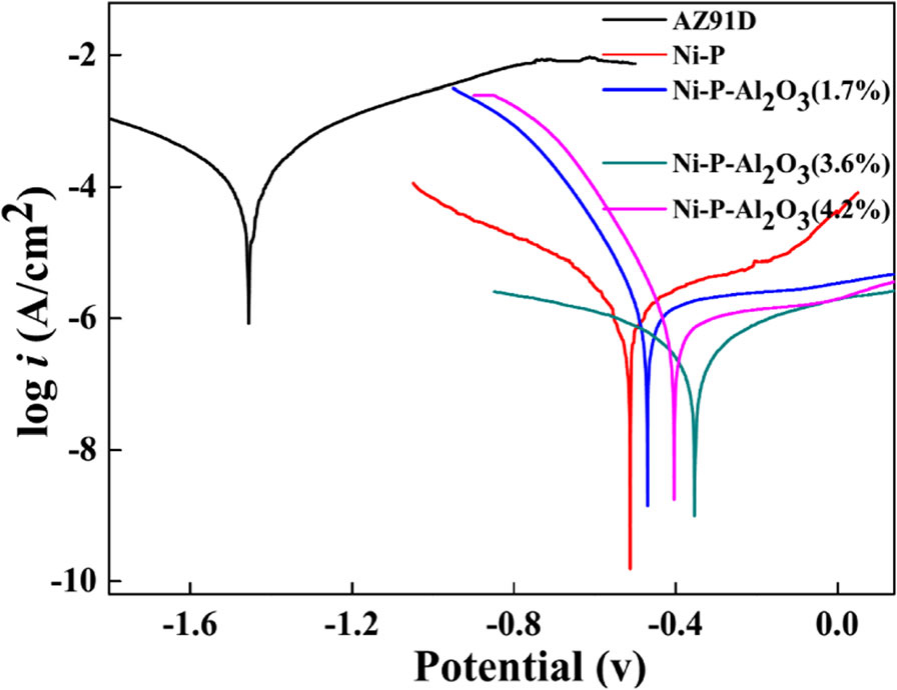
Polarization curves of the AZ91D substrate, the Ni-P coating, and the Ni-P-Al_2_O_3_ composite coatings

Figure 4 and Table 2 show the polarization curves and anti-corrosion parameters of AZ91D Mg alloy substrate, Ni-P coating, and Ni-P-Al_2_O_3_ composite coatings in a 3.5 wt% NaCl aqueous solution at room temperature, respectively. The cathode reaction in the polarization curves corresponds to the hydrogen evolution, while the anodic polarization curves are the most important characteristic reaction processes of corrosion resistance [29]. For the AZ91D Mg alloy substrate, an activation-controlled anodic process is observed when the applied potential increases into the anodic region. Moreover, it is dissolved in electrolyte solution seriously, and its corrosion potential (*E*_corr_) is read at − 1.47 V. But for the *E*_corr_ of the Ni-P coating, it shows a significant positive shift to − 0.51 V compared with that of the Mg alloy substrate (− 1.47 V), and the corrosion current density (*i*_corr_) evidently decreases from 1.4 × 10^−4^ A/cm^2^ of the substrate to 3.1 × 10^−6^ A/cm^2^ of the Ni-P coating (see Table 2). As for Ni-P-Al_2_O_3_ (1.7~4.2 wt%) composite coatings, here, the Al_2_O_3_ content of coatings is obtained by the weighing method. As seen in Table 2, all the *E*_corr_ of the composite coating positive shift and *i*_corr_ of the composite coatings decrease compared with the Ni-P coating, suggesting that Ni-P-Al_2_O_3_ coatings have higher performance in corrosion resistance. Herein, the Ni-P coating with 3.6 wt% of Al_2_O_3_ shows the highest *E*_corr_ (− 0.35 V) and lowest *i*_corr_ (4.5 × 10^−7^ A/cm^2^). However, the *E*_corr_ and *i*_corr_ of Ni-P-Al_2_O_3_ (4.2 wt%) are change to − 0.41 V and 1.0 × 10^−6^ A/cm^2^, respectively. It may be that Al_2_O_3_ particles increase the porosity of Ni-P coating and reduce the performance of composite coatings. Therefore, the Al_2_O_3_ content of composite coatings has an important effect on the corrosion resistance of the composite coatings. It is also related to the structure including crystal plane spacing and grain size of the coatings (Fig. 3).

**Table 2:** Electrochemical corrosion data related to polarization curves of the magnesium alloy, the Ni-P coating, and the Ni-P-Al_2_O_3_ composite coatings

Substrate and coatings	AZ91D	Ni-P	Ni-P-Al_2_O_3_ (1.7%)	Ni-P-Al_2_O_3_ (3.6%)	Ni-P-Al_2_O_3_ (4.2%)
*i*_corr_ (A/cm^2^)	1.4 × 10^−4^	3.1 × 10^−6^	1.6 × 10^−6^	4.5 × 10^−7^	1.0 × 10^−6^
*E*_corr_ (V)	− 1.46	− 0.51	− 0.47	− 0.35	− 0.41

**Fig. 5:**
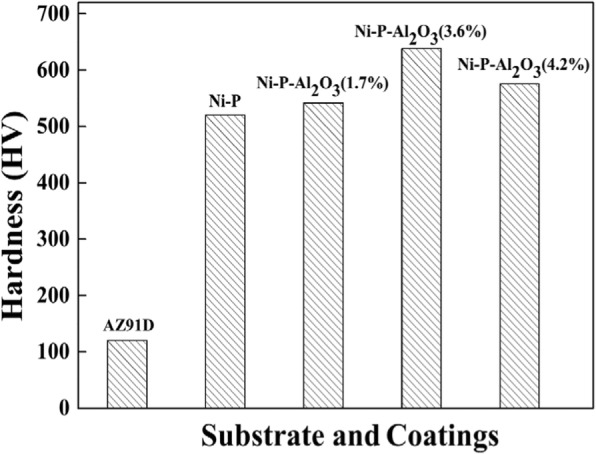
Hardness of the AZ91D substrate, the Ni-P coating, and the Ni-P-Al_2_O_3_ composite coatings

To test the micro-hardness of the coatings, the average thickness of all coatings was determined at 18 μm, which was estimated by the deposition rate and deposition time. The results of micro-hardness tests of Mg alloy substrate and the coatings with different Al_2_O_3_ contents are shown in Fig. 5. As seen in Fig. 5, the micro-hardness of the bare AZ91D Mg alloy is only about 120 HV, whereas the micro-hardness of Mg alloy substrate coated with a Ni-P coating is up to 520 HV. It is higher than the substrate about 400 HV, indicating that Ni-P coating can effectively improve the hardness of the substrate coating. As a result, the wear resistance of Mg alloy substrate is enhanced by the Ni-P coating. Moreover, the Ni-P-Al_2_O_3_ composite coatings show a considerable increase tendency in micro-hardness when the content of Al_2_O_3_ in the coating increases from 0 to 3.6 wt%. Therefore, Ni-P-Al_2_O_3_ (3.6%) composite coatings show the highest hardness value at 638 HV. The reason originates from nano-Al_2_O_3_ particles optimizing the phase structure (see Fig. 3) of the Ni-P alloy and enhancing the micro-hardness of coatings. However, the content of Al_2_O_3_ in the composite coatings reaches 4.2 wt%, and the micro-hardness of coatings decreases to 576 HV instead. This means that higher content of nanoparticles may affect the Ni-P crystal structures leading to unfavorable performance of the composite coatings.

**Fig. 6:**
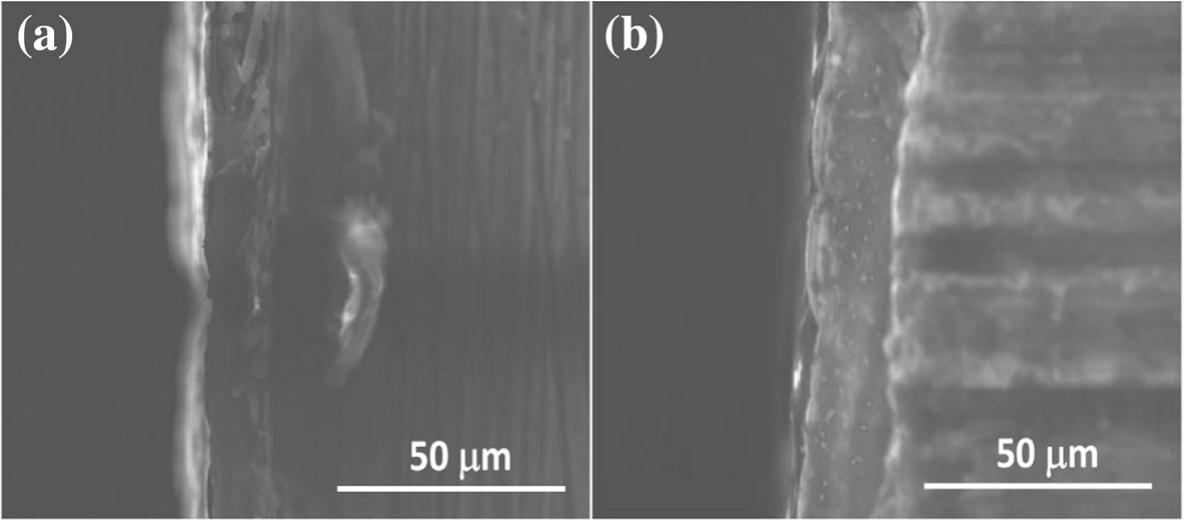
Cross-section morphology images of the Ni-P coating (**a**) and Ni-P-Al_2_O_3_ (3.6 wt%) composite coatings (**b**)

Adhesion between coatings and Mg alloy substrate was carried out by thermal shock test according to the experiment section. Via 20 cycle tests, both the Ni-P coating and Ni-P-Al_2_O_3_ composite coatings well adhered to the Mg alloy substrate. The defects, such as crack, blistering, and spalling, were not observed during the test process, indicating that the Ni-P or Ni-P-Al_2_O_3_ coatings had a good adhesion with the Mg alloy substrate to against the thermal shock process. Moreover, cross-section morphology images between the coatings and Mg alloy substrate were also observed by using SEM. As observed in Fig. 6, it further manifests that there is no apparent defect between the coatings and the substrate via thermal shock test. Importantly, thermal shock test and cross-section observation indicate that nano-Al_2_O_3_ particles have no effect on the adhesion of composite coatings.

**Fig. 7:**
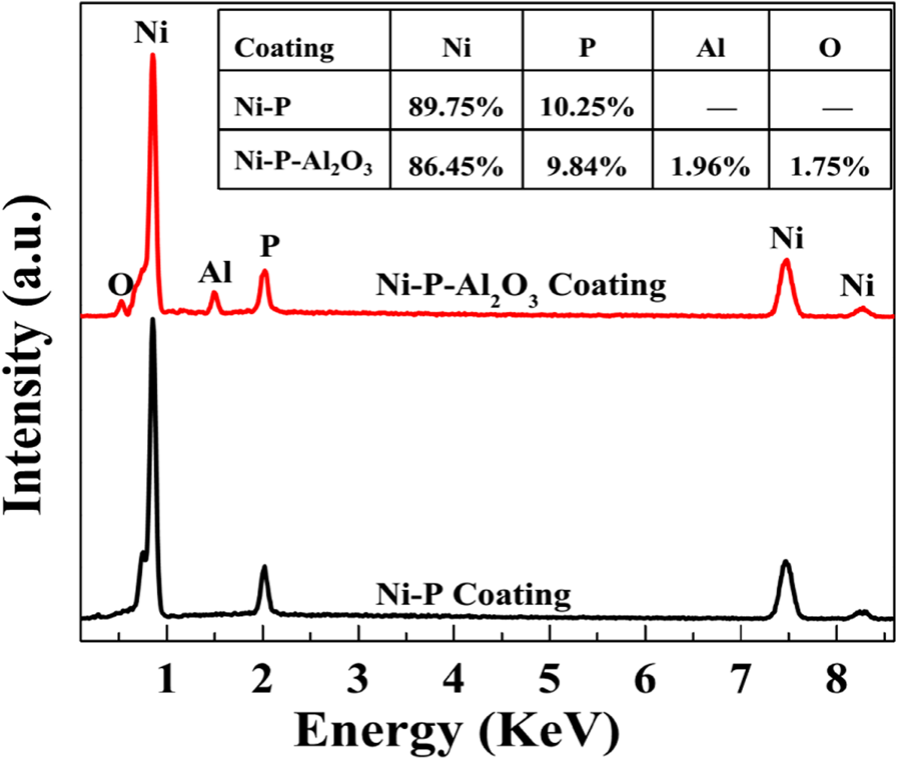
The EDS spectra of the Ni-P coating and the Ni-P-Al_2_O_3_ composite coatings

In the present work, 1-L plating baths without and with nano-Al_2_O_3_ particles (10 g/L) were prepared, respectively. Herein, the initial nickel source content in plating bath was calculated as 7.8 g, and the load capacity of the bath was set at 0.5 dm^2^/L. According to the rules of periodic cycle test (cf. experimental section), the MTO of electroless Ni-P plating bath was firstly evaluated, and about 48.2 g Ni-P alloy was obtained. Here, 90% nickel content was identified in Ni-P coating by using EDS analysis (see Fig. 7). Hence, the content of nickel in the coating can be calculated as 43.4 g. That is, the MTO of plating bath without Al_2_O_3_ particles is 5.6 by using Eq. (2). As for the electroless Ni-P-Al_2_O_3_ composite plating bath, a total of 38.8 g Ni-P-Al_2_O_3_ coatings were deposited from the composite bath. Similarly, 86.45% Ni, 9.84% P, 1.96% Al, and 1.75% O were determined by EDS analysis (Fig. 7). Therefore, the content of nickel in the composite coatings can be calculated as 33.5 g, and the MTO of the composite bath is 4.2. From the results of periodic cycle test, the service cycle of the composite plating bath is 1.4 MTO less than that of the electroless Ni-P plating bath. It means that nano-Al_2_O_3_ particles reduce the service life of electroless plating bath. Nevertheless, the Ni-P-Al_2_O_3_ composite plating still has potential application in the field of magnesium alloy.

## Conclusions

In summary, we obtained an electroless composite plating bath and operating conditions to co-deposit the Ni-P-Al_2_O_3_ coatings on magnesium alloy, i.e., 35 g/L NiSO_4_⋅6H_2_O, 35 g/L lactic acid, 30 g/L Na_2_H_2_PO_2_⋅H_2_O, 10 g/L NH_4_HF_2_, 10 g/L nano-Al_2_O_3_ particles, 3 mg/L stabilizing agent, and pH = 6.0~6.5, *T* = 85 °C, and stirring speed at 350 rpm. Morphology characterization and phase structure analysis of the composite coatings demonstrated that nano-Al_2_O_3_ particles had an important influence on the growth process and phase structures (crystal plane spacing and grain size) of the coatings. 3.6 wt% Al_2_O_3_ content effectively improved the micro-hardness and corrosion resistance of the Ni-P coating. In addition, adhesion test showed that there was almost no difference between Ni-P coating and Ni-P-Al_2_O_3_ coating. Service life test identified the MTO of electroless composite plating bath was about 4. In a word, electroless Ni-P-Al_2_O_3_ composite plating is an important technology to expand the application of magnesium alloy.

## Abbreviations

*E*_0_: Open circuit potential
*i*_corr_: Corrosion current density
Mg: Magnesium
MTO: Metal turnover
Ni-P: Nickel phosphorus
SEM: Scanning electron microscopy
XRD: X-ray diffraction
